# Correlation of biochemical markers and clinical signs of hyperandrogenism in women with polycystic ovary syndrome (PCOS) and women with non-classic congenital adrenal hyperplasia (NCAH)

**Published:** 2012-07

**Authors:** Diana Chanukvadze, Jenara Kristesashvili, Nana Kvashilava

**Affiliations:** 1 *Department of Gynecology, Obstetrics and Reproductology, Medical Faculty, I. Javakhishvili Tbilisi State University, Tbilisi, Georgia. *; 2 *Zhordania Institute of Human Reproduction, Tbilisi, Georgia.*; 3 *Hormonal Laboratory of Zhordania Institute of Human Reproduction, Tbilisi, Georgia. *

**Keywords:** *Hirsutism*, *Hyperandrogenemia*, *Free androgen index (FAI)*, *Polycystic ovary syndrome (PCOS)*, *Non-classic congenital adrenal hyperplasia (NCAH)*

## Abstract

**Background:** Polycystic ovary syndrome (PCOS) is the most common cause of hyperandrogenism in women. Non-classic congenital adrenal hyperplasia (NCAH) is very close to PCOS. The diagnosis of hyperandrogenism is not based on the finding of decreased or increased levels of a single hormone.

**Objective:** In our paper, we are going to test correlation between clinical signs and biochemical markers of hyperandrogenism.

**Materials and Methods:** In this prospective study, we calculated free testosterone (cFT), bioavailable testosterone (cBT), free androgen index (FAI), free estrogen index (FEI), total testosterone (TT), sex-hormone binding globulin (SHBG), estradiol (E_2_), dehydroepiandrosterone-sulfat (DHEA-S), 17α -hydroxyprogesterone (17α -OHP), prolactin (P), C-peptid and homeostasis model assessment for insulin resistance (Homa-IR) were measured in two groups of young untreated women with PCOS and NCAH.

**Results:** In our research, we did not find any significant differences between PCOS and NCAH groups by age, hormonal and calculated parameters of androgens. Waist to hip ratio (WHP) and body mass index (BMI) values were higher in the group of patients with PCOS than NCAH group. But in all patients we found positive correlation between hirsutism score and FAI, cFT, cBT, as well as we found negative correlation between hirsutism score and SHBG. We also tested hormonal and calculated parameters of androgens between PCOS patients by upper body and lower body obesity, but we did not find any significant differences. There was not any difference by the hirsutism score in these groups either.

**Conclusion:** In our research we found that the calculated values of cFT, cBT and FAI are helpful for determinate hirsutism score in all hirsute patients, despite of ovarian or adrenal hyperandrogenemia.

## Introduction

Androgen excess is one of the most common endocrine disorder of reproductive-age women and it affecting approximately 7% of these target population (1, 2, 3).

In women, hyperandrogenism manifested with varying severity and it affects the sebaceous glands (hirsuitism, acne, and alopecia) and ovulatory function (oligoamenorrhea, ovulatory dysfunction and infertility). It can also be as forerunner of other metabolic disorders, such as hyperinsulinemia and dyslipidemia. The appropriate clinical and biochemical assessment of hyperandrogenism in the women helps to have adequate management during the treatment of this disorder (4). Although, PCOS is the most common cause of hyperandrogenism in women, other conditions are taken into account as well. 

The presence of PCOS criteria arising from European Society of Human Reproduction and Embryology (ESHRE) and American Society for Reproductive Medicine (ASRM) symposium (1 may, 2003, Rotterdam, Netherlands) in which PCOS was diagnosed if there are two conditions of the following: 1) oligoovulation and/or anovulation, 2) clinical and/or biochemical signs of hyperandrogenism, 3) polycystic ovaries on ultrasound. But in this time, it is important that the other etiologies of hyperandrogenism (non-classic congenital adrenal hyperplasia, androgen-secreting tumor, Cushing syndrome, hyperprolactinemia and hypothyroidism) have to be exclusion. 

The clinical phenotypes of the Congenital adrenal hyperplasia (CAH) shows a range of severity and include a severe form classified as classic CAH or a mild form classified as non-classic or late-onset CAH (NCAH). Where, CAH is characterized by the cortisol deficiency, with or without aldosterone deficiency and androgen excess. On the other hand, patients with NCAH do not have cortisol deficiency and symptoms of androgen excess, which generally manifested later in childhood or in early adulthood. The symptoms of NCAH include early pubarche, hirsutism (60%), oligomenorrhea or amenorrhea with polycystic ovaries (54%), and acne (33%) (5, 6). This disease is very close to PCOS; therefore, in a patient presenting with phenotypic features of PCOS, it is important to consider NCAH in the differential diagnosis for adequate treatment management.

Compared with other endocrinopathies, the diagnosis of hyperandrogenism is not based on the finding of either decreased or increased levels of a single hormone. Many laboratory markers or clinical signs are used in the evaluation of androgen production. In this case, the most often reported parameters are the level of free testosterone (FT) and the free androgen index (FAI). However, increased levels of androstenedione or other androgen precursors are the only finding in many patients (7). 

It was considered that the measuring of free testosterone (FT) by equilibrium dialysis either calculated free testosterone (cFT), calculated bioavailable testosterone (cBT) from total testosterone (TT), sex hormone–binding globulin (SHBG) and albumin, or the free androgen index (FAI), were more sensitive methods for assessment hyperandrogenemia in women with PCOS and hirsutism (8, 9). All the above mentioned, it is important to continue study in this direction and introduction these methods in clinical practice.

The aim of our study was to investigate correlations between clinical signs and biochemical markers of hyperandrogenism, such as cFT, cBT, FAI those calculated from TT and SHBG, in young women with PCOS and NCAH. We also investigated the comparisons between biochemical markers of hyperandrogenism in upper body and lower body obesity in PCOS patients. 

## Materials

The present prospective study was approved by ethics committee of the Zhordania Institute of Human Reproduction in Georgia. The study population consisted of 59 women in age 13-30 years referred to our clinic from February 2010 to March 2011 for hirsutism, and/or recurrent acne and irregular menstrual cycle. The average age of patients was 17.3 (±3.0). One of the main principles of our sampling was to choose women who were past at least 2 years after the menarche. They did not take any medication, including oral contraceptives, for at least previous 6 months. We had two groups of patients. First group with patients (n=30) having PCOS, fulfilled the diagnostic criteria for PCOS (Rotterdam 2003) and second group with NCAH (n=29) patients diagnosed by high level of 17α-OHP. 

All of the patients underwent a complete physical examination including measurement of height, weight, waist and hip circumference, body mass index (BMI) [weight (kg) divided by height square (m^2^)] and the waist to hip ratio (WHR) as the ratio between the waist and the hip circumferences were calculated. 

Blood was sampled to measure serum androgens. Blood samples were assayed at 8:00-10:00 am, during 3^rd^-5^th^ day of menstrual cycle (only follicular phase). The morphology of the ovaries was observed by pelvic or transvaginal ultrasound scans (Siemens Sonoline G 50). Hirsutism was evaluated according to Ferriman and Gallwey scores in 9 sites of body (10). 

Each site was scored 0-4 points based on the severity of hirsutism. On this basis, total scores of 8 and over were considered as hirsute and below this range as nonhirsute (11). Calculations of cFT and cBT were performed using the formula available on the web site of the International Society for the Study of an Aging Male (ISSAM) (http://www. issam.ch/freetesto.htm), from TT and SHBG measured in the same sample from each woman. This method has been described in detail by Vermeulen *et al* (12). FAI was obtained as the quotient: 100* (TT/SHBG) (13). The computer based HOMA-2 calculator (available at www. dtu. ox. ac. uk/ homa) uses fasting glucose and C-peptid to generate the homeostasis model assessment for insulin resistance (HOMA-IR). 

All assays were conducted in our hormonal laboratory using established commercial assays routinely monitored by participation in external quality-control programs. Total testosterone (TT) and estradiol were measured with Enzyme-linked immunosorbent assay (ELISA, DRG, Diagnostic Products Corporation Germany). The calibration range of the TT assay was 0.2-16 ng/ml, with an analytical sensitivity of 0.083 ng/ml. The cross-reaction with 11-β hydroxyestosterone was 3.3%, 19-Nortestosterone 3.3%. The calibration range of the estradiol assay was 25 pg/ml-2000 pg/ml, with an analytical sensitivity of 9.714 pg/ml. The cross-reaction with estriol and estron 0.05%, 0.2% respectively. 

Sex hormone-binding globulin (SHBG), DHEAS and 17α -hydroxyprogesterone were measured with Enzyme-linked immunosorbent assay (ELISA, IBL, Diagnostic Products Corporation Germany).The calibration range of the DHEAS assay was 0.1-10 µg/ml, with an analytical sensitivity 0.004 µg/ ml. The cross-reaction with androsteron-sulfat 5.67%. The calibration of SHBG assay was 4-260 nmol/l, with an analytical sensitivity 0.2 nmol/l. No cross -reactivity with other compounds was known. The calibration range of the 17-OH-Progesterone assay was 0.15-20 ng/ml, with an analytical sensitivity 0.03 ng/ ml. 

The cross-reaction with 17α-OH-Pregnenolone 1.7%, Progesterone 1.4%, 11-Desoxy-Cortisol 1.3%. Prolactin was measured with Enzyme-linked immunosorbent assay (ELISA, Human, Diagnostic Products Corporation Germany). The calibration range of prolactin assay was 5-100 ng/ml, with an analytical sensitivity 0.8 ng/ml. No cross -reactivity with other compounds was known. C-peptid was measured with Enzyme-linked immunosorbent assay (ELISA, Novatec Diagnostic Products Corporation Germany). The calibration range of the C-peptid assay was 0.2-10 ng/ml, with an analytical sensitivity 0.025 ng/ml at the 95% confidence limit. The cross-reaction with Proinsulin 1.2%. 

**Statistical analysis**

All data were analyzed with SPSS software (statistical Package for the Social Sciences, version 17.0 for windows XP; SPSS, Inc, Chicago, I17). A p-value of less than 0.05 was considered to be statistically significant. 

Two-group comparison of continuous variables was performed using a two-sample ''*t *test'' with adjustment for non constancy of variance, when necessary. More than two group means were compared using the ANOVA with post hoc*.* Correlations between continuous variables were analyzed with Pearson’s correlation. 

## Results

The clinical, anthropometric, hormonal and metabolic variables in the 30 patients in PCOS and 29 patients in NCAH groups are presented in table I. There are no significant difference by age, TT, cFT, cBT, SHBG, E_2_, FEI, DHEA-S, C-peptid and Homa-IR values (p>0.05). WHR and BMI values were significantly elevated in PCOS patient compare to NCAH group (p<0.05). 

Prolactin level and 17α -OHP were significantly higher in NCAH group (p<0.05). Women with PCOS had higher FAI value in comparison with women with NCAH, but not significantly. The patients with PCOS (100%) and patients with NCAH (55.2%) had oligomenorrhea. Hirsutism was defined in 80% patients with PCOS and 75.9% in patients with NCAH.

**Comparison of PCOS hirsute women with NCAH hirsute women**

PCOS hirsute women in comparison with NCAH hirsute women had significantly higher BMI, WHR and FEI value. Some biochemical markers of hyperandrogenemia such as FAI, cFT and cBT were higher in PCOS hirsute women, but not significantly. On the other hand, TT and DHEAS were higher in NCAH hirsute women, but not significantly. 

No differences were found in hirsutism score and SHBG level between PCOS and NCAH groups (Table II).

**Comparison of different degrees of hirsutism with biochemical markers of hyperandrogenemia**

We divided all our patients into three groups by different degrees of hirsutism, which was scored in accordance with the modified Ferriman-Gallwey score. Minimal hirsutism by 3-7 points, mild hirsutism by 8-16 points and moderate or severe hirsutism- over 17 points were assessed. In our study, we used the analysis of variance (ANOVA) comparing three groups of patients with hirsutism to each other. 

There was significantly high value of FAI and cBT between minimal and moderate hirsutism patients. There was no significant difference with regard to TT, SHBG, cFT and DHEAS, but there was tendency of decrease SHBG in comparison with hirsutism degree (Table III).

**Correlations between hirsutism degree and various anthropometric, hormonal parameters and calculated biochemical markers of hyperandrogenemia**

Considering all patients together we found positive correlations between hirsutism score and FAI, cFT, cBT as well as negative correlation with SHBG (Table IV).

**Comparison of upper and lower body obesity with biochemical markers of hyperandrogenemia, insulin resistance and hirsutism score**

With regard to WHR, we divided PCOS patients into two groups using value of WHR of 0.80 as cutoff: into upper-body obesity (WHR >0.80) and lower body obesity (WHR ≤0.80). Patients with upper-body obesity had significantly high Homa-IR and high FEI (p<0.05), than those with lower body obesity. 

PCOS patients with upper body obesity in comparison with patients with lower body obesity had lower SHBG and higher FAI, but not significantly. No differences were obtained in hirsutism score between groups (Table V).

**Table I:** Clinical, anthropometric, hormonal and metabolic characteristics between PCOS and NCAH patients groups

**Characteristics**		**PCOS (n=30)**	**NCAH (n=29)**	**p-value**
Age (years)		17.3±3.5	17.5±2.4	0.817
Body mass index (BMI)		27.2±7.9	21.7±3.3	0.001
Waist to hip ratio (WHR)		0.82±0.08	0.76±0.06	0.002
Total Testosterone (TT)		0.67±0.31	0.79±0.32	0.152
Sex-hormone binding globulin (SHBG)		27.6±23.5	30.5±27.2	0.663
Free androgen index (FAI)		6.0±7.3	4.4±3.5	0.273
Calculated free testosterone (cFT)		2.4±0.9	2.2±0.8	0.426
calculated bioavailable testosterone (cBT)		56.6±21.7	51±17.5	0.273
C-peptid		1.9±1.0	2±0.8	0.947
Homeostasis model assessment for insulin resistance (Homa-IR)		1.4±0.7	1.4±0.6	0.759
Dehydroepiandrosterone sulfat (DHEA-S)		2.4±1.4	2.8±1.5	0.472
17α-hydroxyprogesterone (17α -OHP)		0.76±0.22	1.5±0.5	0.001
Estradiol (E_2_)		33.9±23.6	30.1±22.6	0.647
Free estrogen index (FEI)		0.3±0.3	0.1 ±0.1	0.070
Prolactin		11.9±4.0	18.2±11.9	0.012
TSH		2.2±1.1	2.3±1.3	0.789
Menstrual cycle pattern [n (%)]				
	Regular cycle	0 (%)	13 (44.8%)	
	Oligomenorrhea	30 (100%)	16 (55.2%)	
Hirsutism				
	Present	24(80%)	22 (75.9%)	
	Absent	6 (20%)	7 (24.1%)	

PCOS: Polycystic ovary syndrome.

NCAH: Non-classic congenital adrenal hyperplasia.

* Note: Results are expressed as the mean±SD. p-value consider significant when it <0.05.

**Table II:** Comparison of PCOS hirsute women with NCAH hirsute women anthropometric and biochemical markers of hyperandrogenemia.

	**PCOS with hirsutism (n=24)**	**NCAH with hirsutism (n=22)**	**p-value**
Body mass index (BMI)	26.3±6.8	21.9±3.6	0.008
Waist to hip ratio (WHR)	0.81±0.08	0.75±0.06	0.010
Hirsutism score	16.4±5.6	16.2±5.4	0.886
Total Testosterone (TT)	0.7±0.33	0.81±0.33	0.214
Sex-hormone binding globulin (SHBG)	25.2±23.8	27.4±21.7	0.743
Free androgen index (FAI)	7.0±7.9	4.7±3.8	0.211
Calculated free testosterone (cFT)	2.5±1.0	2.2±0.7	0.317
Calculated bioavailable testosterone (cBT)	60±21.8	52±16.7	0.178
Free estrogen index (FEI)	0.32±0.3	0.11±0.05	0.010
Dehydroepiandrosterone sulfat (DHEA-S)	2.3±1.5	3.0±1.6	0.364

Note: Results are expressed as the mean±SD.

* p-value consider significant when it<0.05.

**Table III:** Clinical, anthropometric, hormonal and metabolic characteristics between different degrees of hirsutism

**Characteristics**	**3-7 points (n=13)**	**8-16 points (n=26)**	**17 and more points (n=20)**	**p-value**
Body mass index (BMI)	25.4±9.1	23.1±5.0	25.7±6.7	0.376
Waist to hip ratio (WHR)	0.78(±0.08	0.77±0.08	0.8±0.08	0.440
Total Testosterone (TT)	0.64±0.27	0.73±0.34	0.78±0.32	0.468
Sex-hormone binding globulin (SHBG)	38.9±31.9	29±26.4	22.6±16.4	0.195
Free androgen index (FAI)	2.9±2.3*	4.7±3.8	7.4 ±8.4*	0.030
Calculated free testosterone (cFT)	1.9±0.8	2.3±0.9	2.4±0.8	0.251
Calculated bioavailable testosterone (cBT)	45.4±18.3*	53.7±20.6	59.5±18.5*	0.048
Estradiol (E_2_)	27.9±17.5	32.9±25.2	34.2±24.2	0.847
Free estrogen index (FEI)	0.16±0.18	0.18±0.19	0.33±0.31	0.205
Dehydroepiandrosterone sulfat (DHEA-S)	2.3±1.3	2.3±1.5	2.3±1.5	0.109
Prolactin	17.6±12.4	13.4±5.0	15.2±10.9	0.408
TSH	1.6±1.1	2.4±1.2	2.2±1.1	0.217

Note: Results are expressed as the mean ± SD.

* p-value consider significant when it <0.05.

**Table IV:** Correlations between Ferriman-Gallwey score of hirsutism and various anthropometric, hormonal parameters and calculated biochemical markers of hyperandrogenemia

**Charecteristics**	**r**	**p-value**
Age (years)	-0.072	0.585
Body mass index (BMI)	0.038	0.777
Waist to hip ratio (WHR)	0.076	0.567
Total Testosterone (TT)	0.138	0.299
Free androgen index (FAI)	0.312	0.016
Calculated free testosterone (cFT)	0.288	0.027
Calculated bioavailable testosterone (cBT)	0.315	0.015
Sex-hormone binding globulin (SHBG)	-0.313	0.016
Dehydroepiandrosterone sulfat (DHEA-S)	0.290	0.086
Estradiol (E_2_)	0.115	0.519
Free estrogen index (FEI)	0.322	0.063

* p-value consider significant when it <0.05.

**Table V:** Difference between group of lower - body obese and upper - body obese PCOS in biochemical marker of insulin resistance and hyperandrogenemia

**Characteristics**	**Lower-body obese PCOS (n=14)**	**Upper-body obese PCOS (n=16)**	**p-value**
Body mass index (BMI)	21.6±2.7	32.1±7.6	0.001
Total Testosterone (TT)	0.65±0.37	0.67±0.27	0.880
Sex-hormone binding globulin (SHBG)	35.3±28.1	20.8±16.6	0.106
Free androgen index (FAI)	4.2±4.0	7.6±7.6	0.197
Calculated free testosterone (cFT)	2.1±1.0	2.5±0.8	0.254
Calculated bioavailable testosterone (cBT)	53.3±24.3	59.5±19.5	0.445
Free estrogen index (FEI)	0.1±0.1	0.4±0.3	0.014
Homeostasis model assessment for insulin resistance (Homa-IR)	0.9 ±0.4	1.6±0.8	0.007
Hirsutism score (by Ferriman-Gallwey)	13.3±4.9	14.8±8.5	0.564

Note: Results are expressed as the mean ± SD

* p-value consider significant when it <0.05.

## Discussion

In this study we investigated biochemical markers for assessment of clinical signs of hyperandrogenemia in women with PCOS and with NCAH for adequate treatment management. Women were strictly classified as having PCOS according to the revised Rotterdam 2003 diagnostic criteria. Recently, the Androgen Excess Society suggests that the original National Institutes of Health criteria should be accepted with a few modifications, defining PCOS as an androgen excess syndrome or “hyperandrogenic syndrome” (14). Hirsitism in women is a clinical expression of hyperandrogenism, it is one of the most widespread diagnostic endocrine disorder in young women and affects approximately 5-10% of women (15). 

In our study the presence or grade of increased body hair was chosen as a clinical marker of hyperandrogenism. This is well correlated with biochemical markers of hyperandrogenemia such as FAI, cFT and cBT. The results of Pearson's test demonstrated significant positive correlation of FG-score with cFT, cBT and FAI as well as significant negative correlation of the Ferriman-Gallwey score with SHBG. This is an important argument supporting the essential role of androgen production in the clinical manifestation increase of body hair. In our study we did not find any difference in hirsutism scores by Ferriman- Gallwey between PCOS hirsute and NCAH hirsute patients. In PCOS hirsute group we found higher FAI value, then in NCAH hirsute group, but not significantly. 

On the other hand, in NCAH hirsute group we found higher DHEA-S and TT, then in PCOS hirsute group, but not significantly. This fact can explain why we did not find any difference between these groups by hirsutism score. Free testosterone (FT) is the most prevalent marker in women with androgen excess, but the measurement procedures for FT are not routinely practicable in many laboratories. Some models have been developed for calculating FT from total testosterone, sex-hormone binding globulin and albumin. In general, previous studies show that, these calculations gave good correlation with equilibrium dialysis of FT and considered to be a reliable indicator of FT. No differences were observed when we measured only simple enzyme immune-assays of TT, DHEAS and SHBG. That is why only these parameters are not effective for evaluation of hirsutism. 

In this study we also divided patients with PCOS into two groups by value of waist to hip ratio (WHR), if WHR >0.8 we assume that patients had upper-body obesity and WHR ≤0.8 had lower-body obesity. We did not find differences between these two groups by biochemical markers of hyperandrogenemia, but there were tendency to increased FAI and decreased SHBG in the patients with upper body obesity. Hirsutism score was similar in both groups. We found significantly high value of FEI in upper body obesity patients. It is well known that increase of body weight and fat tissue is associated with several abnormalities of sex steroid balance.

Such alterations involve androgens, estrogens and their carrier protein SHBG. Changes in SHBG concentrations lead to alteration of androgen and estrogen delivery to target tissues. In fact, female patients with upper body obesity usually have lower SHBG concentrations in comparison with their age and weight-matched counterparts with lower body obesity (16). Obesity can also be considered as a condition of increased estrogen production, the rate which correlates significantly with body weight and the amount of body fat. Reduced SHBG concentrations may in turn lead to increased exposure of target tissues to free estrogens (17). 

Approximately 50-70% of women with the condition known as PCOS were described as showing hyperinsulinemic insulin resistance (IR), which may play a major role in the development of the PCOS (18). Women with hyperandrogenic syndrome are an ideal group for identifying insulin resistance (IR) at an early stage and preventing its complications later in life (19). A close relationship between hyperandrogenism, hyperinsulinemia and IR was reported from studies in females with elevated androgen levels due to PCOS (20). In our study we decided to compare patients with PCOS and NCAH by Homa-IR, but we did not find any difference by Homa-IR index between patients with PCOS and NCAH. 

This fact can be explain that hyperandrogenism is an independent risk factor for hyperinsulinism in women and might have a role in the development of insulin resistance or polycystic ovaries in patients with NCAH (21, 22). According our study we can concluded that the calculated values of cFT, cBT and FAI are helpful for determinate hirsutism score in all hirsute patients, despite of ovarian or adrenal hyperandrogenemia.
